# Optogenetic excitation of cholinergic inputs to hippocampus primes future contextual fear associations

**DOI:** 10.1038/s41598-017-02542-1

**Published:** 2017-05-24

**Authors:** Sarah Hersman, Jesse Cushman, Noah Lemelson, Kate Wassum, Shahrdad Lotfipour, Michael S. Fanselow

**Affiliations:** Department of Psychology and Psychiatry, University of California, Los Angeles, California, 90095 USA

## Abstract

Learning about context is essential for appropriate behavioral strategies, but important contingencies may not arise during initial learning. A variant of contextual fear conditioning, context pre-exposure facilitation, allows us to directly test the relationship between novelty-induced acetylcholine release and later contextual associability. We demonstrate that optogenetically-enhanced acetylcholine during initial contextual exploration leads to stronger fear after subsequent pairing with shock, suggesting that novelty-induced acetylcholine release primes future contextual associations.

## Introduction

During exploration of a novel context, the dorsal hippocampus (DH) must integrate highly processed, sequential sensory inputs into a configural contextual representation, which may take on associative valence, or be stored for later recall^1^. Cholinergic inputs to DH from the medial septum, in particular, have been associated with both memory encoding and contextual processing^2^ and increase with exploration of a novel environment^3^, suggesting their essential role in regulating these processes. However, important contextual contingencies, such as an encounter with a threat, often do not occur with the first experience of a context. An open question is how signaling during novel context exposure could facilitate later contextual learning, when the context is familiar and neuromodulators such as acetylcholine (ACh) are no longer as elevated.

**Figure 1:**
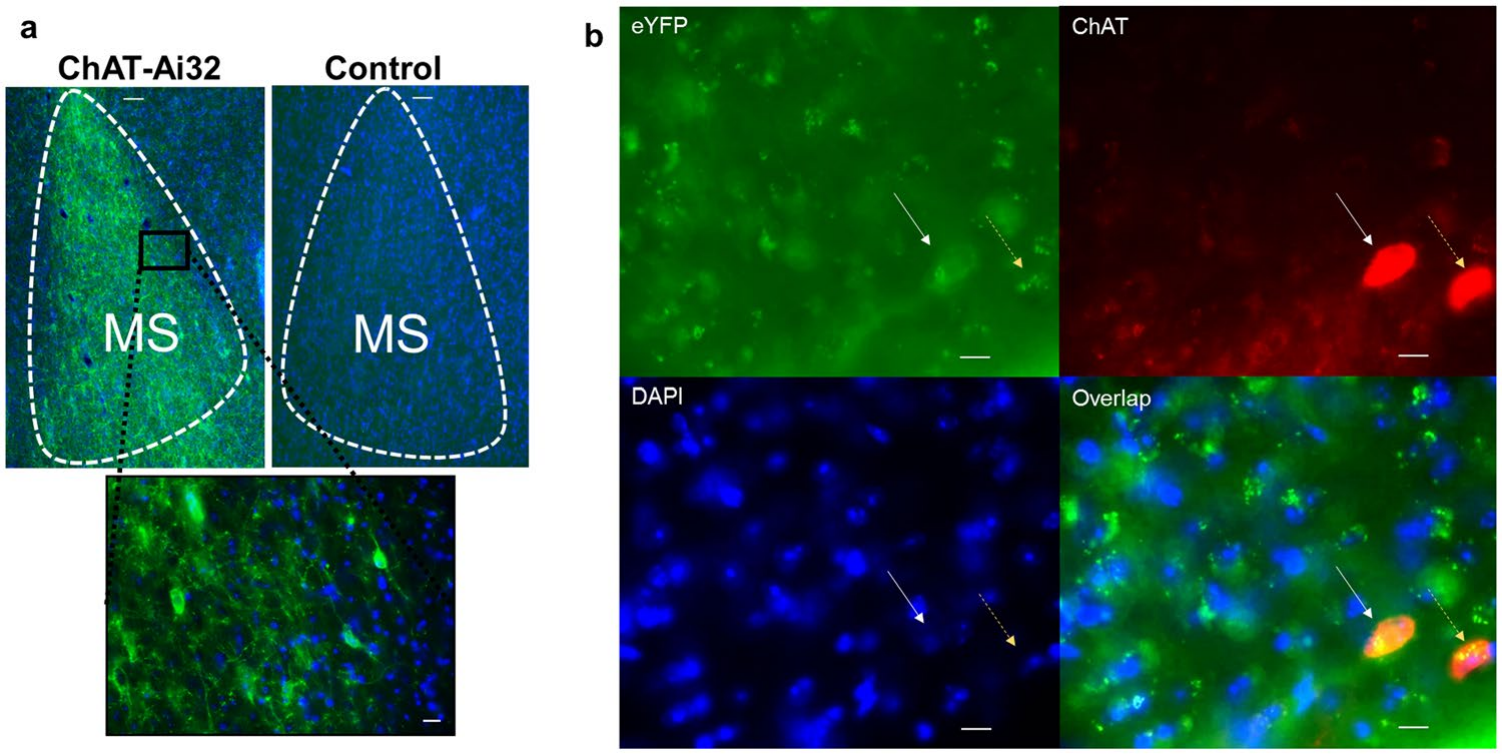
ChAT-Ai32 mice expressed eYFP in a subset of cells in MS, as well as in other forebrain regions. No fluorescence was visible in littermate controls negative for Cre. (**a**) Slices (40 μm) were taken from ChAT-Ai32 mice and littermate controls negative for Cre and imaged using light microscopy (scale bars top 50 μm, bottom 10 μm). Nuclei are stained with DAPI. (**b**) A subset of slices were immunostained for ChAT to demonstrate co-localization with native eYFP in ChAT-Ai32 mice (white arrow eYFP+, yellow arrow eYFP−, scale bars 10 μm).

To model this in mice we used the Contextual Pre-exposure facilitation Effect (CPFE) procedure to separate across days this initial learning about context and later pairing with an aversive stimulus, to determine if elevated ACh during initial exploration might affect later contextual fear. We selectively increased ACh release by optogenetically stimulating cholinergic cell bodies in medial septum in ChAT-Ai32 mice^4, 5^ and Ai32 littermate controls (Fig. 1). Previous studies^6^ have demonstrated very high (~100%) co-localization between ChAT and YFP expression, indicating robust and selective transgene expression in medial septal cholinergic neurons. In addition, we validated and characterized our *in vivo* optogenetic manipulation using choline biosensors to measure light-evoked acetylcholine release in dCA1 of anesthetized mice.

**Figure 2:**
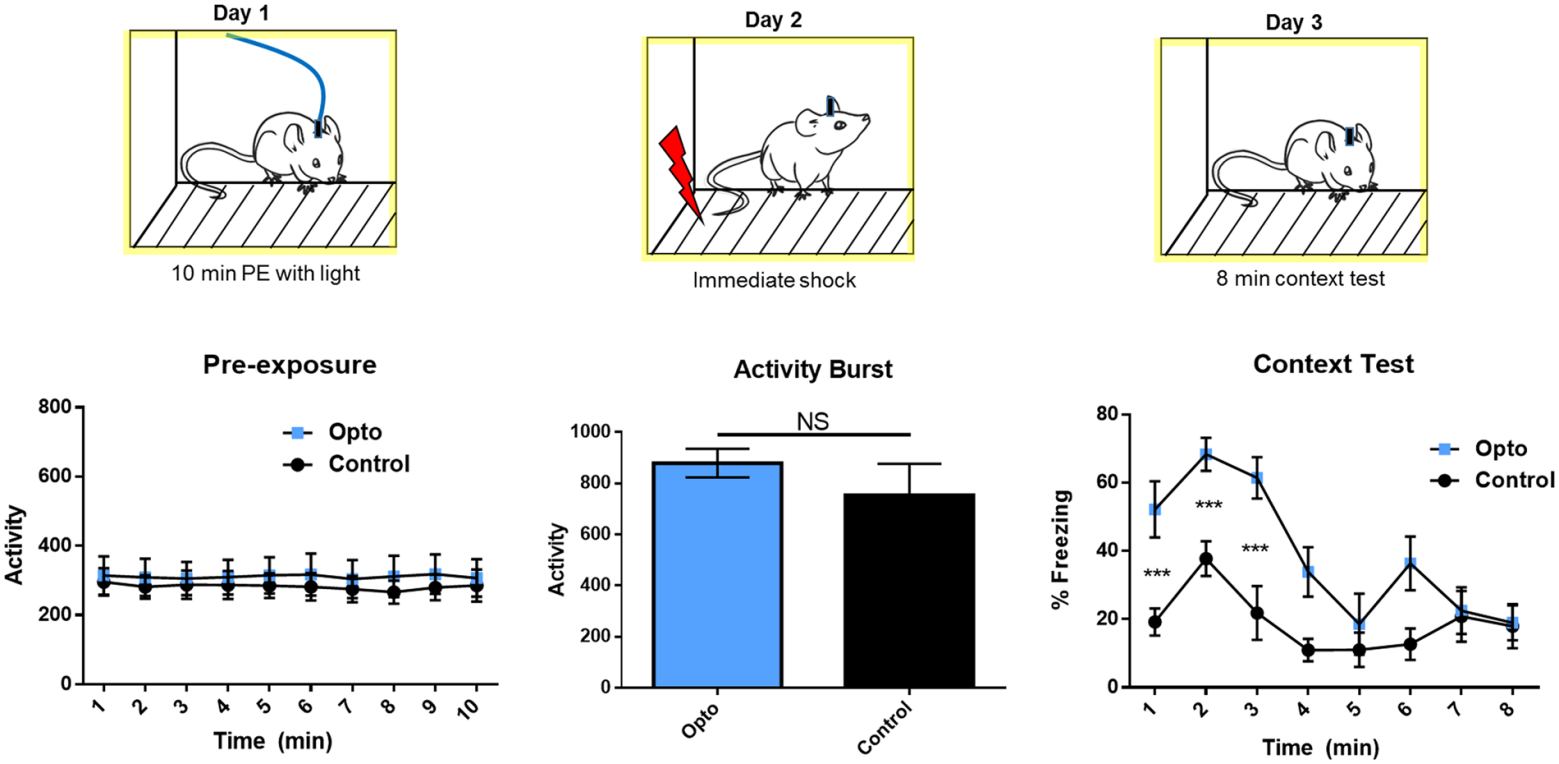
The behavioral protocol is shown above the results from each session. Mice were exposed to a novel context during optogenetic stimulation of medial septum cholinergic neurons (n = 6 Opto and n = 10 Controls); activity did not differ between groups during preexposure (PE) (p > 0.05). The following day, mice were shocked immediately (10 sec) after placement in the context (n = 6 per group); no difference was observed in freezing before or after shock (data not shown) nor in activity burst to the shock (p > 0.05). Opto mice demonstrated significantly greater freezing the following day at test (p < 0.05).

During novel context exploration, ChAT-Ai32 mice and littermate controls (opto and control) received blue light stimulation. On Day 2, mice were returned to the same context for ten seconds before receiving a foot shock, which is sufficient time for a rodent to recall a previously-formed contextual representation, but not sufficient for novel encoding^7^. Thirty seconds after the shock, they were removed. Mice were tested for fear in an 8-min context exposure on Day 3 (Fig. 2). Optically-evoked acetylcholine release was verified in a separate group of subjects (See Supplementary Fig. 1).

**Figure 3:**
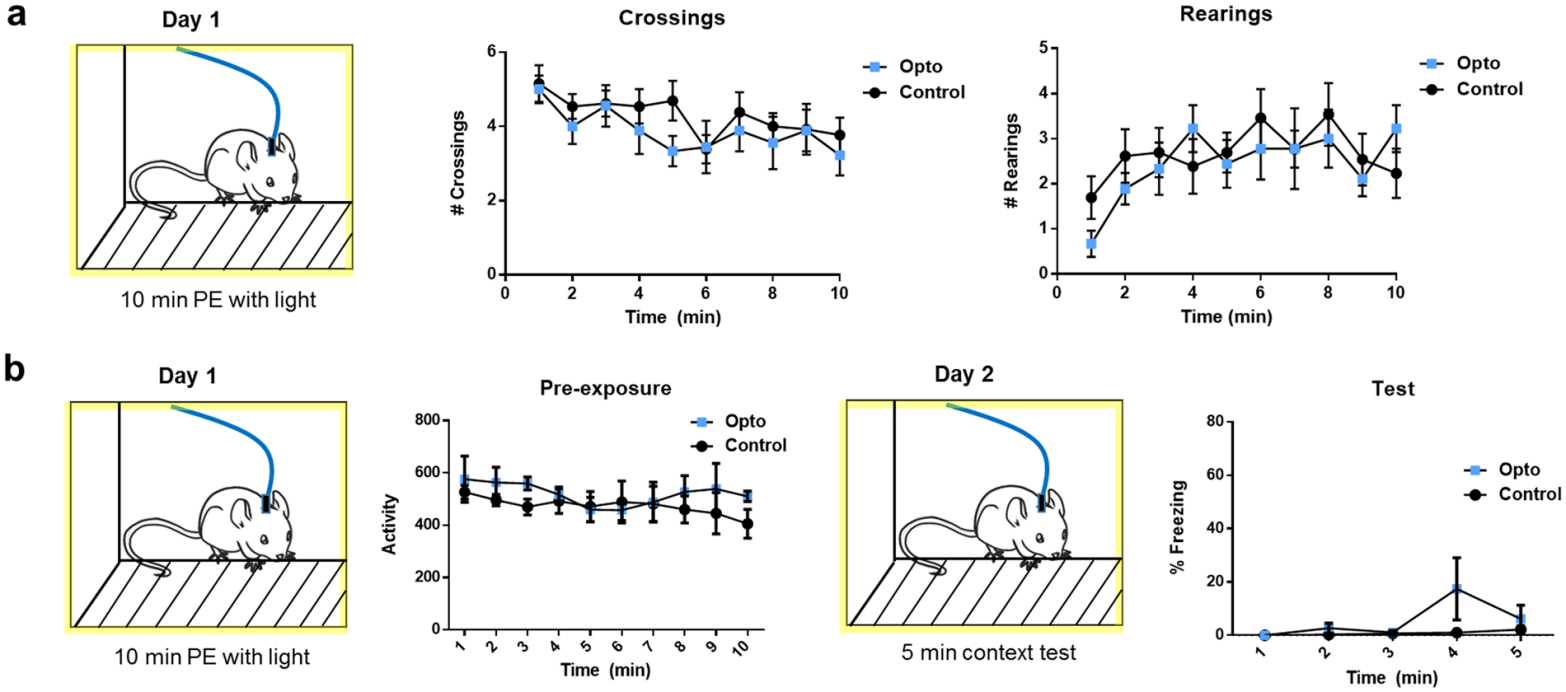
The behavioral protocol is shown to the left of the results from each session. (**a**) No effect of optogenetic enhancement of ACh release was observed on rearings or crossings (hand-scored) during stimulation (n = 9 opto, n = 13 control, p > 0.05). (**b**) A separate cohort was given optogenetic stimulation (n = 3 opto, n = 6 control) during pre-exposure (PE) and tested for fear behavior the following day. There was no difference in activity under stimulation (p > 0.05), and no freezing or group differences were observed at test (p > 0.05).

Higher levels of acetylcholine are correlated with increased exploratory drive^3, 8^, leading to the possibility that cholinergic signaling promotes exploration. This would manifest as greater sampling of contextual details, and could promote a more detailed context representation. However, this was not the mechanism for our effect. We found that stimulating cholinergic release did not change overall exploratory activity (F(1, 11) = 0.2, p > 0.5) nor did exploration habituate throughout the ten minute exposure at the population level in either group (Greenhouse-Geisser corrected Repeated Measures ANOVA, F(4.6, 64.5) = 0.614, p > 0.5; no interaction between session time and genotype (p > 0.5)). In addition, we hand-scored behavior during light stimulation, in order to quantify crossings and rearings, specific behavioral actions required for complete contextual sampling (Fig. 3a). Neither crossings nor rearings differed between groups (p > 0.05), suggesting that while the release of ACh has been shown to correlate with exploration, optogenetic stimulation of ACh release does not drive increased exploratory behavior in a novel context. This may be due to the fact that exploratory drive is already high in a novel context, or that cholinergic release is not the driving force behind behavioral exploration. Pharmacological blockade of muscarinic receptors does not alter exploratory activity^9^, which favors the interpretation that ACh signaling does not direct behavioral exploration.

Increased cholinergic activity during contextual encoding led to a profound enhancement in fear when that context was later paired with shock. Though no between-group differences were observed in freezing in the pre-shock or post-shock periods during acquisition on the second day (p > 0.05), opto mice froze significantly more during the context test (Repeated Measures GLM, Main Effect of Genotype, F(1, 10) = 8.805, p < 0.05). This effect was greatest during the first three minutes of the context test, before both groups underwent within-session extinction of the fear response (Greenhouse-Geisser corrected, Main Effect of Time, F(2.66, 26.5) = 16.82, p < 0.0001, Time X Genotype Interaction, F(2.66, 26.5) = p < 0.01, Sidak-Bonferroni corrected t-tests on minutes 1–3 (p < 0.005)). This difference suggests that increased cholinergic tone during contextual encoding primes a context for future associations, which manifests as increased fear after the context is paired with shock.

Other possibilities we addressed were that enhanced cholinergic activity during learning somehow alters sensitivity to future sensory stimuli such as shock, or that the stimulation itself is aversive, and simply sums with the negative valence due to shock to lead to higher levels of fear at test. Importantly, there were no changes in responsivity to shock (activity burst, p > 0.05), which rules out a change in sensitivity to shock as a result of cholinergic enhancement. We tested this second possibility by repeating our behavioral procedure without Day 2, to determine if freezing would occur to the context without pairing with shock (Fig. 3b). No freezing was detected, nor were there differences in overall activity between groups on either day (p > 0.05), suggesting that the stimulation itself was not acting as an innately aversive US. Therefore, the enhanced fear we observed at test in our main cohort was due to pairing an aversive shock with a primed contextual association, leading to enhanced encoding of that association.

In order to test the functional consequence of MS cholinergic optogenetic stimulation, choline biosensors were used to record optogenetically-evoked ACh in DH. Evoked ACh scaled with laser power and pulse width, and stimulation protocols used in our behavioral experiments led to sustained elevation of release (Supplementary Fig. 1). These data demonstrate that a pulse train that evokes electrochemically-measurable choline also evokes cognitive changes, suggesting that these measured elevations are on a behaviorally relevant scale. Anesthetized recording of optogenetically-evoked ACh in the hippocampus indicated that our optogenetic stimulation parameters approximated the magnitude of naturally evoked transients recorded in awake rats in the prefrontal cortex^10^.

These behavioral data provide evidence that cholinergic signaling controls processing of contextual information in the hippocampus. Specifically, increasing cholinergic inputs, above the release normally produced by exposure to a novel environment, primes a context for future associability with relevant stimuli. These behavioral effects are consistent with electrophysiological recordings under increased ACh conditions, as optogenetic activation of cholinergic neurons enhanced theta band oscillations and suppressed competing frequency bands such as sharp-wave ripples^6^, which may provide a mechanistic explanation for enhancements observed here. Future studies will need to address the limitations and tradeoffs inherent in priming future associations, such as the erroneous associations that may occur in similar contexts.

## Materials and Methods

### Subjects

Twenty-three naïve male ChAT-Ai32 mice, aged 4–5 months of age and weighing 25–30 g were singly housed post-surgery and maintained on a 12-hour light/dark cycle with access to food and water *ad libitum*. Animals were handled for five days leading up to behavioral experiments. 6 naïve female ChAT-Ai32 mice, aged 5 months and weighing 25 g, were used for non-survival anesthetized choline biosensor recordings. The behavioral and surgical procedures used in this study were in accordance with policy set and approved by the Institutional Animal Care and Use Committee of the University of California, Los Angeles.

### Apparatus

Behavioral training used fear conditioning chambers (30 × 25 × 25 cm, Med-Associates, Inc St. Albans, VT), equipped with a Med-Associates VideoFreeze system. The boxes were enclosed in larger sound-attenuating chambers in an individual, dedicated experimental room. The context was comprised of a chamber with aluminum sidewalls and a white Plexiglas rear wall. The grid floor consisted of 16 stainless steel rods (4.8 mm thick) spaced 1.6 cm apart (center to center). The ceiling was clear Plexiglas with a central hole allowing for passage of fiberoptic cables. Pans underlying each box were sprayed with a thin film of 50% Windex solution to provide the context with a scent. Chambers were individually lit from above by white lights and cleaned with 50% Windex in between trials. Fans mounted above each chamber provided background noise (60 dB). The experimental room was brightly lit with an overhead white light. Animals were kept in a holding room and individually transported to the experimental room in their home cage. On the first day of training, animals were transported to the habituation cart for cable attachment before conditioning, and returned to the cart for cable disconnection afterward. Chambers were cleaned with a Virkon solution following each day of behavioral testing.

### Surgery for *In Vivo* Optogenetics

Mice were anesthetized with 3% isoflurane, and standard surgical procedures were used to implant a single fiberoptic ferrule cannula targeting the medial septum, with these coordinates (AP: +0.70, ML: 0.00, DV: −3.5). Cannula were fixed in place using one layer of Metabond (Parkell, Inc.), and a second layer of dental acrylic (The Bosworth Company). Mice were allowed to recover for at least one week before undergoing behavioral training. At the end of experiments, mice were perfused and placement in MS was confirmed (Supplementary Fig. 2a).

### Context Pre-exposure Procedure

Animals received two days of transport to holding room and attachment to the fiberoptic cable (5 min per animal, per day) prior to the experiment. On the second day, animals were pre-exposed to the LED light stimulation for use during behavior (470 nm, 5 mW, 10 Hz, 5ms pulses) for 5 min. For context pre-exposure, animals were transported in home cage to the habituation cart, where they were briefly restrained and connected to the fiberoptic cable. After a 2 min habituation period, animal was transported to the context chamber, where 10 min LED light stimulation and contextual exposure took place. Mice were then removed to habituation chamber, and after 2 min were disconnected and returned to their home cage. The following day, mice were transported in the home cage to the same context chamber, were after 10 seconds they received a foot shock (0.75 mA, 1 sec); 30 sec later they were removed and returned to home cage. The following day, mice were transported in the home cage to the same context chamber, where freezing was recorded for 8 min.

### Acetylcholine Recording

#### Summary

Briefly, these biosensors use choline oxidase as the biological recognition element and rely on electro-oxidation, via constant-potential amperometry (0.7 V versus a Ag/AgCl reference electrode), of enzymatically-generated hydrogen peroxide (reporter molecule) to provide a current signal. This current output is recorded and converted to choline concentration using a calibration factor determined *in vitro*. Choline sensing allows for an accurate extracellular measure of acetylcholine, which is rapidly hydrolyzed by endogenous acetylcholinesterase^11–14^. Indeed, adding acetylcholinesterase onto the sensing electrode does not enhance detection of cholinergic activity^12^. Interference from both electroactive anions and cations is effectively excluded from the amperometric recordings, while still maintaining a <1-s response time, by application of polymer coatings to the electrode sites prior to enzyme immobilization^15^. Additionally, incorporation of two non-enzyme-coated sentinel electrodes on the MEA enabled removal of correlated noise from the choline sensing electrode output by signal subtraction (see Data Analysis). Completed sensors were sealed in a container with desiccant and stored at 4 °C.

#### Reagents

Nafion (5 wt % solution in lower aliphatic alcohols/H_2_O mix), bovine serum albumin (BSA, min 96%), glutaraldehyde (25% in water), pyrrole (98%), choline chloride (99%) L-ascorbic acid, 3-hydroxy-tyramine (dopamine) were purchased from Aldrich Chemical Co. (Milwaukee, WI, USA). CholOx with a rated activity of 10 units per mg protein (U mg-1, Lowry’s method) was purchased from Sigma (Sigma-Aldrich Co., St. Louis, MO, USA). Phosphate buffered saline (PBS) was composed of 50 mM Na 2 HPO 4 with 100 mM NaCl (pH 7.4). Ultrapure water generated using a Millipore Milli-Q Water System was used for preparation of all solutions used in this work.

#### Instrumentation

Microelectrode array (MEA) probes were fabricated in the Nanoelectronics Research Facility at UCLA and modified for choline detection. Electrochemical preparation of the sensors was performed using a Versatile Multichannel Potentiostat (model VMP3) equipped with the ‘p’ low current option and low current N’ stat box (Bio-Logic USA, LLC, Knoxville, TN). *In vitro* and *in vivo* measurements were conducted using a low-noise multichannel Fast-16 mkIII potentiostat (Quanteon), with reference electrodes consisting of a glass-enclosed Ag/AgCl wire in 3 M NaCl solution (Bioanalytical Systems, Inc., West Lafayette, IN) or a 200 μm diameter Ag/AgCl wire, respectively. All potentials are reported versus the Ag/AgCl reference electrode.

#### Sensor Preparation and Calibration

Biosensors prepared for choline detection were calibrated *in vitro* to test for sensitivity, selectivity and response time to choline. Coating layers on the sensors used in the study were as follows: one sensor with PPY and three sensors with PPD; all sensors also contained a Nafion coating layer. *In vitro* was carried out using constant potential amperometry with the FAST-16 electrochemistry system. A constant potential of 0.7 V was applied to the working electrodes against a Ag/AgCl reference electrode in 40 mL of stirred PBS at pH 7.4 and 37 °C within a Faraday cage. Data were collected at 80 kHz and averaged over 1 s intervals. After the current detected at the electrodes equilibrated to baseline (approx. 30 min), an aliquot of the potential interferents, AA (250 µM final concentration; representative potential anionic interferent) and DA (5–10 µM final concentration; representative potential cationic interferent) were added to ensure selectivity for choline. For all sensors used in these experiments no current responses to these interferents were detected above the level of the noise. The sensors were calibrated against three 40 µL aliquots of choline (20 mM), which were added to the beaker to reach a final choline concentration of 20, 40 and 60 µM choline. Hydrogen peroxide (10 µM final concentration) was also added, to ensure similar sensitivity and response time on control and choline-sensitive channels. Average H_2_O_2_ sensitivity for the sensors used in the study varied no more than 10% between control and choline sensor electrodes and was not significantly different (ttest, p > 0.05). A representative calibration can be found in the supplement (Supplementary Fig. 1). A calibration factor based on analysis of these data was calculated for each electrode on the biosensor to be used for *in vivo* anesthetized experiments. Data were output as current as a function of time and analyzed in Microsoft Excel.

### *In Vivo* Validation of Optically-Evoked Acetylcholine Release

For all biosensor experimental animals included in the study, we attempted to stimulate release of ACh in DH using a fiber implanted in MS. We did not see significant changes in signal. This is likely due to two factors. The first is that the size of the biosensor in DH necessitated using a 20 degree angle for the fiber placement in the MS, likely limiting the portion of the MS illuminated by the light to a subset of the dorsal portion. The second is that this subset of MS cells may not have mapped directly to the CA1 region where our biosensor was placed, reducing or eliminating the signal observed. Therefore, we directly stimulated axon terminals close to the biosensor to maximize our chances of targeting the correct MS axons. Therefore, a biosensor was implanted into DH and a ferrule delivering blue light was inserted near the biosensor. Standard stereotaxic surgical techniques under isoflurane anesthesia were used to unilaterally implant a biosensor, pre-calibrated to choline (see above) into the dorsal hippocampus (dCA1), following coordinates according to the atlas of Paxinos and Watson (4th ed.) (AP: −1.95, ML: +1.25, DV: −1.25 to −1.5). Additionally, a fiberoptic ferrule cannula (Doric Lenses) was implanted, angled at 20 degrees, into dCA1 (AP: −1.95, ML: +0.2, DV: −1.6 to −1.8). A Ag/AgCl reference electrode was implanted in contralateral prefrontal cortex. The entire experiment was conducted inside a Faraday cage. The probe was advanced to the pyramidal layer of dCA1 and the electrode signal was allowed to equilibrate to baseline for approximately 30 min prior to application of 3 s light pulses at 10 mW. Successive pulses of light and advancing of the fiberoptic occurred by 0.1 to 0.2 mm each time until the maximal current response was evoked on the biosensor. When a response was consistently observed, all mice received the following protocol with three repetitions of each. First, a power titration (1.5 s pulses at 1, 2, 3, 5, 10, 20 mW), followed by a pulse width titration (5 mW power, 1000 ms, 500 ms, 250 ms, 125 ms with continued halving until no visible response was seen). Then a strong pulse train (5 mW, 1 s on 0.5 s off for 45 s). Finally, a test of 10 Hz pulse trains (5 mW, first 5 ms, then 10 ms, then 20 ms pulses, for 10 min). Stimulations were administered at least 30 s apart, with longer baseline periods for longer pulse trains. All data were plotted as current versus time using GraphPad Prism (La Jolla, CA) and SPSS (IBM Corp, Chicago, IL). At the conclusion of the recording session, brains were removed and placement of fiber and biosensor in DH was confirmed (Supplementary Fig. 2b).

### Statistical Analysis

For contextual fear conditioning, statistical analyses on freezing behavior were performed using an automated near infrared (NIR) video tracking equipment and computer software (VideoFreeze, Med-Associates Inc.), as previously described^16^. For biosensor recordings, currents are reported as calibrated changes in choline concentration; each stimulation was repeated at least three times. The current changes from baseline on the control electrode were subtracted from current changes on the choline biosensor electrode to remove noise correlated among the electrodes on the MEA; this also removed the light induced-electrical artifact, which was similar across channels. The baseline subtraction for shorter (<30 s) stimulation protocols was taken from an averaged 10 s bin 20 s before the onset of light stimulation. For longer stimulation protocols (<30 s), the baseline subtraction was taken from an averaged 10 s bin 1 min before the onset of light stimulation. The choline biosensor response then was converted to choline concentration using an electrode-specific calibration factor obtained *in vitro*, which averaged 54.95 μM/nA. For all hypothesis tests, the α level for significance was set to p < 0.05. The data were analyzed with paired t-tests, repeated-measures ANOVAs (with post hoc analysis correcting for multiple comparisons), correlation and regression, where appropriate.

## Electronic supplementary material

Supplementary Information
