# Supplementary material for: Optogenetic excitation of cholinergic inputs to hippocampus primes future contextual fear associations
Source: Sci Rep. 2017 May 24;7:2333. doi: 10.1038/s41598-017-02542-1 (PMC5443779; doi:10.1038/s41598-017-02542-1)
Supplement: Supplementary file 1 — Supplementary Information [file 41598_2017_2542_MOESM1_ESM.pdf]

Supplementary Information

Optogenetic excitation of cholinergic inputs to hippocampus primes future contextual fear associations.

Sarah Hersman, Jesse Cushman, Noah Lemelson, Kate Wassum, Shahrdad Lotfipour & Michael S. Fanselow.

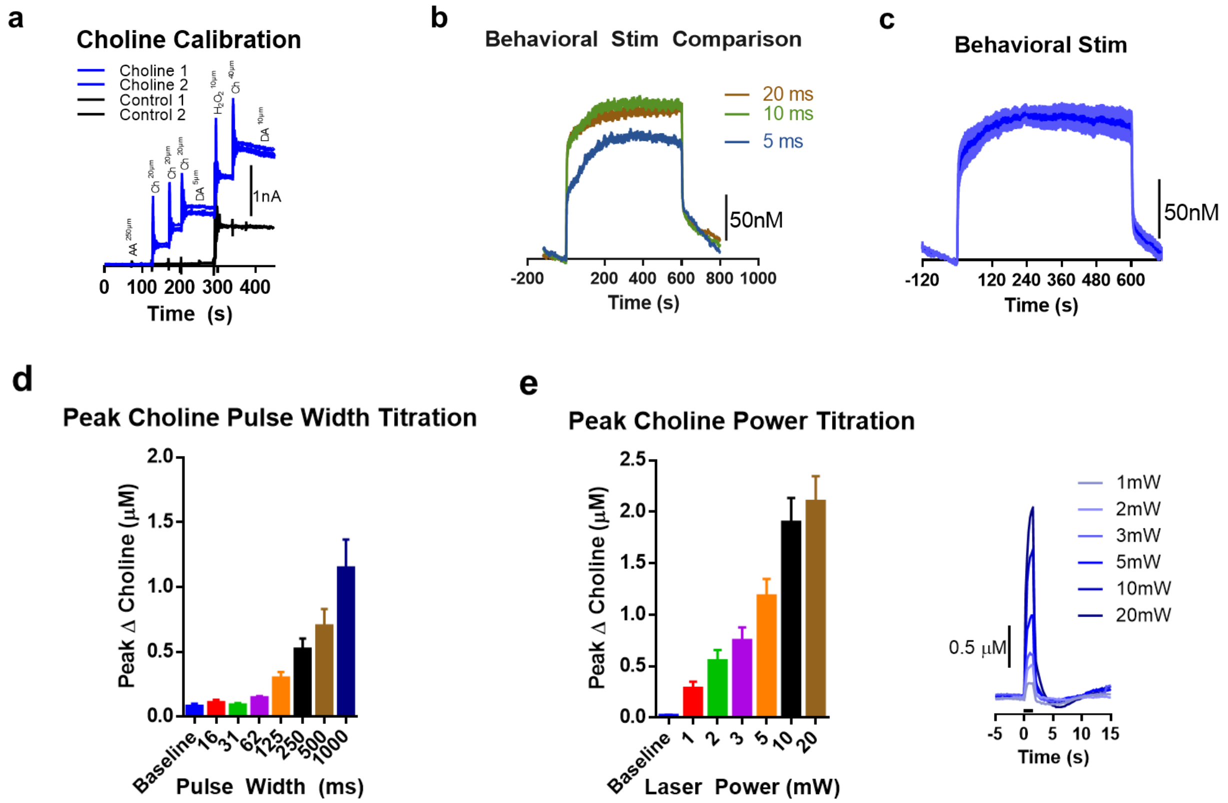

Supplementary Figure 1 Choline biosensor recording of optically-evoked acetylcholine from ChAT-Ai32 mice. (**a**) Representative calibration from a choline sensor used in the study. (**b**) Single representative traces of stimulation parameters tested for use during behavior (all at 5mW, 5ms pulse width, 10Hz). Power and pulse width were chosen as being representative of mild stimulation protocols used in other recent optogenetic experiments[16] while stimulation frequency was chosen to exceed slightly the highest recorded firing rate of putative MS cholinergic neurons in vivo, which was measured at 4-6Hz[17]. The lowest measurable and sustained trace was chosen for behavior (5ms). (**c**) Average evoked acetylcholine during pulse train chosen for behavior (10 min, 5mW, 10Hz, 5ms pulse. Standard error of 7 traces shown here: 2 mice, 4 sensors). (**d**) Summary of the maximal Ach evoked at multiple pulse widths (5mW power). Evoked Ach scales with pulse width in all subjects, and single 62ms pulses and above are significantly elevated above baseline ($p<0.05$) (individual data not shown). (**e**) Summary of the maximal Ach evoked at multiple laser power levels (1.5s pulse). Individual representative trace of evoked ACh during power titration shown at right.

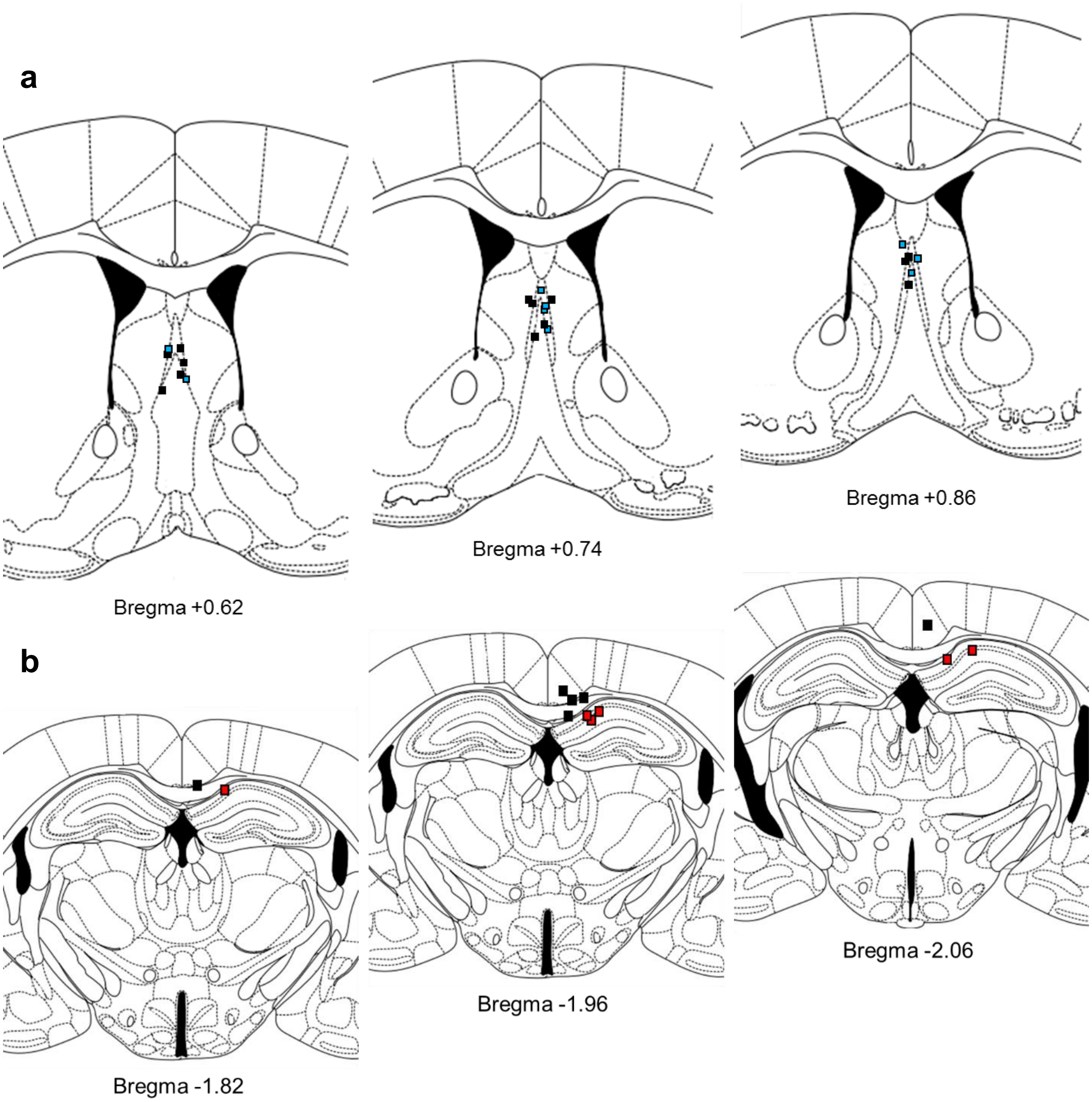

Supplementary Figure 2 (**a**) Fiber tip placements for behavioral experiments (Control, black; Opto, blue). (b) Fiber tip and biosensor tip placements for anesthetized recording experiments (Fiber, black; Biosensor, red).

Supplementary References

16. Mamad, Omar, Harold M. McNamara, Richard B. Reilly, and Marian Tsanov. “Medial Septum Regulates the Hippocampal Spatial Representation.” *Frontiers in Behavioral Neuroscience* 9 (June 30, 2015). doi:10.3389/fnbeh.2015.00166.

17. Zhang, Hao, Shih-Chieh Lin, and Miguel A. L. Nicolelis. “A Distinctive Subpopulation of Medial Septal Slow-Firing Neurons Promote Hippocampal Activation and Theta Oscillations.” *Journal of Neurophysiology* 106, no. 5 (November 1, 2011): 2749–63. doi:10.1152/jn.00267.2011.
